# *Echinacea*-induced cytosolic Ca^2+ ^elevation in HEK293

**DOI:** 10.1186/1472-6882-10-72

**Published:** 2010-11-23

**Authors:** Lankun Wu, Eric W Rowe, Ksenija Jeftinija, Srdija Jeftinija, Ludmila Rizshsky, Basil J Nikolau, Jodi McKay, Marian Kohut, Eve Syrkin Wurtele

**Affiliations:** 1 Department of Genetics, Development, and Cell Biology, Iowa State University, Ames, IA, 50011, USA; 2 Center for Research on Dietary Botanical Supplements at Iowa State University and the University of Iowa, Ames, IA, 50011, USA; 3 Department of Biomedical Sciences, Iowa State University, Ames, IA, 50011, USA; 4 Department of Biochemistry, Biophysics, and Molecular Biology, Iowa State University, Ames, IA, 50011, USA; 5 Department of Biology and Chemistry, Morningside College, Sioux City, IA 51106, USA; 6 Department of Kinesiology, Iowa State University, Ames, IA, 50011, USA

## Abstract

**Background:**

With a traditional medical use for treatment of various ailments, herbal preparations of *Echinacea *are now popularly used to improve immune responses. One likely mode of action is that alkamides from *Echinacea *bind to cannabinoid type 2 (CB2) receptors and induce a transient increase in intracellular Ca^2+^. Here, we show that unidentified compounds from *Echinacea purpurea *induce cytosolic Ca^2+ ^elevation in non-immune-related cells, which lack CB2 receptors and that the Ca^2+ ^elevation is not influenced by alkamides.

**Methods:**

A non-immune human cell line, HEK293, was chosen to evaluate *E. purpurea *root extracts and constituents as potential regulators of intracellular Ca^2+ ^levels. Changes in cytosolic Ca^2+ ^levels were monitored and visualized by intracellular calcium imaging. U73122, a phospholipase C inhibitor, and 2-aminoethoxydiphenyl borate (2-APB), an antagonist of inositol-1,4,5-trisphosphate (IP_3_) receptor, were tested to determine the mechanism of this Ca^2+ ^signaling pathway. *E. purpurea *root ethanol extracts were fractionated by preparative HPLC, screened for bioactivity on HEK293 cells and by GC-MS for potential constituent(s) responsible for this bioactivity.

**Results:**

A rapid transient increase in cytosolic Ca^2+ ^levels occurs when *E. purpurea *extracts are applied to HEK293 cells. These stimulatory effects are phospholipase C and IP_3 _receptor dependent. *Echinacea*-evoked responses could not be blocked by SR 144528, a specific CB2 receptor antagonist, indicating that CB2 is not involved. Ca^2+ ^elevation is sustained after the *Echinacea*-induced Ca^2+ ^release from intracellular Ca^2+ ^stores; this longer-term effect is abolished by 2-APB, indicating a possible store operated calcium entry involvement. Of 28 HPLC fractions from *E. purpurea *root extracts, six induce cytosolic Ca^2+ ^increase. Interestingly, GC-MS analysis of these fractions, as well as treatment of HEK293 cells with known individual and combined chemicals, indicates the components thought to be responsible for the major immunomodulatory bioactivity of *Echinacea do not *explain the observed Ca^2+ ^response. Rather, lipophilic constituents of unknown structures are associated with this bioactivity.

**Conclusions:**

Our data indicate that as yet unidentified constituents from *Echinacea *stimulate an IP_3 _receptor and phospholipase C mediation of cytosolic Ca^2+ ^levels in non-immune mammalian cells. This pathway is distinct from that induced in immune associated cells via the CB2 receptor.

## Background

Well known for its characteristic fiery and pungent taste, *Echinacea *produces local anesthesia of the mucous membranes; thus, it has been used medicinally since ancient times [1]. *Echinacea *was used by Native Americans as a remedy to treat a number of ailments; principally in relation to the alleviation of pain and the promotion of healing, in cases of snake bites, burns, cough, sore throats, and toothache [1]. *Echinacea *products are currently promoted as general enhancers of the immune system, and are among the top-selling herbal preparations in the U.S.A [2]. Despite the popularity of *Echinacea *as an herbal supplement, and many pharmacological and clinical studies, the molecular mechanisms of action for *Echinacea *are not well understood. Among the phytochemicals that accumulate in *Echinacea *[2,3], four major classes, polyunsaturated alkamides/ketones, caffeic acid derivatives, glycoproteins, and polysaccharides, exhibit biological effects *in vitro *and *in vivo*. These include anti-inflammatory, anti-fungal, anti-viral, and immunostimulatory activities [1,4,5]. However, it is uncertain which specific compound(s) are primarily responsible for these bioactivities, or whether they are efficacious in humans [4]. Furthermore, the early molecular events associated with cellular exposure to *Echinacea *are unknown.

Recently, a possible mode of action for *Echinacea *was proposed based on indications that some alkamides bind to cannabinoid (CB) receptors *in vitro *[6,7]; the concentrations of alkamides required for binding to the CB2 receptor, as reported by different groups, have differed by as much as 30-fold (60 nM, [6]; 2-20 μM, [7]). More recently, these alkamides, as well as crude *E. purpurea *extracts, were reported to transiently induce intracellular calcium (Ca^2+^) levels in HL60 cells via CB2 receptor activation [8].

Because of Ca^2+^'s central role as a key intracellular second messenger mediating diverse range of cellular processes [9], and because so little is understood about the early events associated with *Echinacea-*induced bioactivity, we postulate that metabolic components from this genus may induce intracellular Ca^2+ ^increase, which could mediate a series of physiological process involved in this bioactivity.

To evaluate the possibility that *Echinacea *components impact Ca^2+ ^homeostasis, the effects of applying *Echinacea *extracts and HPLC-purified fractions from these extracts were studied in HEK293 cells using intracellular calcium imaging. The HEK293 line was chosen because of its nature as a human non-immune cell line, its well-characterized transcriptome and the apparent absence of CB receptors [10].

## Methods

### Plant Material and Extraction and Preparative HPLC Fractionation

Plant materials were provided by the USDA North Central Regional Plant Introduction Station (NCRPIS, Ames, IA). *E. purpurea *(accession PI631307) was used in all experiments. Further information about this accession can be found on the Germplasm Resources Information Network database at http://www.ars grin.gov/npgs/acc/acc_queries.html.

*Echinacea *extracts were prepared from roots of 2-year-old field grown plants, soxhlet ethanol extraction and preparative HPLC fractionation of the extracts were performed as previously described [11]. All extracts and fractions were dried and re-dissolved in 100% DMSO and diluted 1000 times with HEPES buffer (final concentration of DMSO is 0.1%) before applied to the cells.

### GC-MS Analysis

GC-MS analysis was used to determine concentrations of known alkamides present in *E. purpurea *fractions through the use of synthetic standards as previously described [11].

### Chemicals

Fura-2 AM, pluronic F-127, Dulbecco's modified Eagle's Medium, penicillin-streptomycin, L-glutamine, and trypsin were purchased from Invitrogen (Carlsbad, CA, USA); thapsigargin, 2-aminoethoxydiphenyl borate (2-APB), 1-[6-[((17β)-3-Methoxyestra-1,3,5[10]-trien-17-yl)amino]hexyl]-1H-pyrrole-2,5-dione (U-73122) and 1-[6-[((17β)-3-Methoxyestra-1,3,5[10]-trien-17-yl)amino]hexyl]-2,5-pyrrolidinedione (U-73343) were purchased from Sigma (St. Louis, MO, USA). SR 144528 was generously provided by NIDA Drug Supply System (Baltimore, MD, USA). Alkamide and ketone standards were synthesized as previously described [11].

### Cell Culture

HEK293 cells were obtained from ATCC (Manassas, VA, USA) and cultured in Dulbecco's modified Eagle's Medium supplemented with 10% FBS, 50 U/ml penicillin, 50 μg/ml streptomycin, and 2 mM glutamine. Cells were grown in an incubator at 37°C with humidified 5% CO_2 _and 95% air.

### Intracellular Calcium Imaging

Intracellular calcium concentrations ([Ca^2+^]_i_) were measured by ratiometric imaging techniques using a perfusion system as previously described [12]. Cells were plated onto 22-mm microscope coverslips 36 hr before the experiment. Cells were loaded with Fura 2-AM for 60 min at room temperature. The loading solution contained 1 μl of 25% (w/w) Pluronic F-127 and 4 nM of Fura 2-AM diluted in 1 ml of HEPES buffer. The loading solution was removed and the cells were washed twice with HEPES buffer before the coverslips were placed onto a perfusion chamber and connected to a micro pump. The test chemicals were placed in syringes on a five-valve manifold and added into the perfusion chamber using a micro pump with a flow rate of 200 μl per min. The application lengths of *Echinacea *extracts, fractions and chemicals were indicated by the bars on the graph as well as in the figure legends. As a result of the physical distance between the syringe and the cell chamber, there was a 2 min delay between adding the test or control samples and the exposure of the cells to the samples. Initial analysis of calcium imaging data was conducted using MetaFluor^® ^software. Maximum increase in [Ca^2+^]_i _(Δ[Ca^2+^]_iMax_) was determined as the difference from the resting value to the maximum response. The time-to-peak value was measured and defined as the time it took for the signal to reach from the resting value to the maximum response. The response length was defined as the time interval from the time the signal reaches the maximum response to the time it took to return to the resting value. The dose-response curve was generated using PRISM 4.02 (GraphPad Software, San Diego, CA, US). The half-maximum concentration of *Echinacea *extract that induces a response (EC_50_) was calculated from the fitted sigmoidal curves using PRISM 4.02.

### Statistical Analysis

The cytosolic [Ca^2+^] increase data (Δ[Ca^2+^]_i_) were represented as mean ± SEM, n = 3; data were compiled from cell traces from three biological replicates (three independent cell cultures), measuring at least 20 cell traces/replicate; each value is the mean of ≤ 20 cells from one replicate. Means of Δ[Ca^2+^]_iMax _were analyzed by t-test to determine statistical significance compared to the control. One-way analysis of variance followed by the Tukey test was also carried out to compare means between different treatments. All statistical analyses were performed using SAS software version 9.1 (SAS Institute Inc., Cary, NC).

## Results

### *E. purpurea *extracts induce transient cytosolic [Ca^2+^] increase in HEK293 cells

Roots of *E. purpurea *are among those most widely used medicinally [4], thus we focused on this species, using a well-characterized accession (PI631307) grown and processed under defined conditions [3].

**Figure 1:**
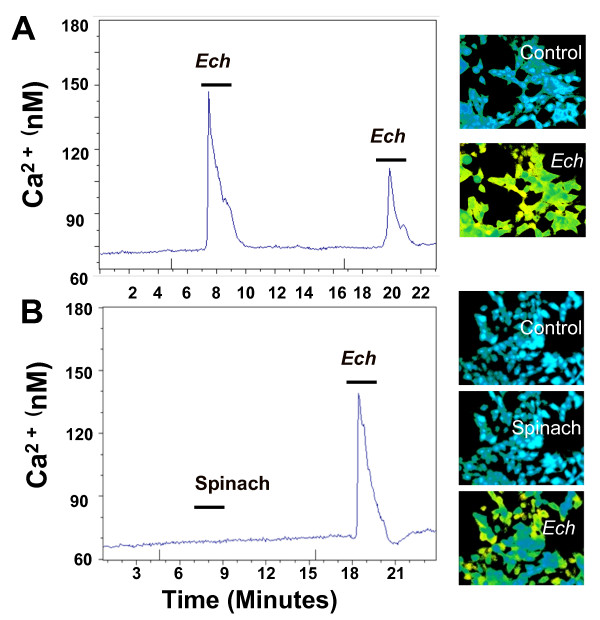
***E. purpurea *root ethanol extracts induce a transient increase in cytosolic Ca^2+ ^concentrations in HEK293 cells**. (A) Left: Real-time monitoring of the change in cytosolic Ca^2+ ^concentrations in HEK293 cells in response to repeated application of *E. purpurea *(*Ech*) extract (100 μg/ml). Δ[Ca^2+^]_iMax _= 71 ± 4 nM (mean ± SEM, *n *= 3). Time-to-peak = 32 ± 2 s (mean ± SEM, *n *= 3). This *Echinacea*-evoked increase in cytosolic Ca^2+ ^concentration was statistically significant (p < 0.001) as compared to the control cells, which were treated with 1% DMSO (dissolved in HEPES buffer).. Right: Pseudocolor images of calcium concentration in cells before treatment (control) and after treatment with *E. purpurea *extract. (B) Left: Real-time monitoring of the change in cytosolic Ca^2+ ^in HEK293 cells in response to application of spinach extract (200 μg/ml), and then a subsequent application of *E. purpurea *extract (100 μg/ml). Right: Pseudocolor images of calcium concentration in cells before treatment (control), after treatment with spinach extract, and after treatment with *E. purpurea *extract. *E. purpurea *and spinach extracts were prepared with 95% ethanol [11], dried, and re-dissolved in 100% DMSO and diluted 1000 times with HEPES buffer (final concentration of DMSO is 0.1%) before applied to the cells for 2 min (the bar in the graph represents the application length). Data is an average trace of the treatment of at least 20 cells in each experiment, representative of three independent experiments.

**Figure 2:**
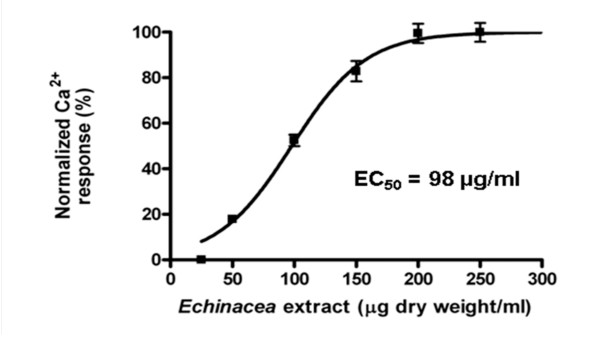
**Dose-response curve of the transient increase in cytosolic Ca^2+ ^concentration in HEK293 cells evoked by *E. purpurea *root ethanol extracts**. Results are expressed as percentages of maximum responses. Each data point represents mean ± SEM, n = 3 replicates. EC_50 _= 98 ± 7 μg/ml. The response saturates at 200 μg/ml of extract. The Hill equation was used to fit the response curve.

*E. purpurea *root ethanol extracts evoke a transient [Ca^2+^]_i _increase in HEK293 cells (Figure 1A). This *Echinacea*-evoked calcium response returns to baseline rapidly after removal of the *Echinacea *extract (Figure 1A), indicating that the activation is reversible. At a dose of 100 μg extract/ml, the increase of [Ca^2+^]_i _was 71 ± 4 nM, and the time-to-peak of this response was 32 ± 2 s. A second application of extract induces a second transient increase in [Ca^2+^]_i _of somewhat less intensity than the first. Similar attenuation of the response to second application of the ligands has been previously demonstrated and was determined to be due to receptor desensitization [13] Similarly prepared ethanol extracts of an non-*Echinacea *species, spinach, were tested as a control, no [Ca^2+^]_i _increase was observed (Figure 1B). To investigate the concentration-dependence of this *Echinacea *response, seven concentrations of extracts, ranging from 25 to 300 μg/ml, were evaluated (Figure 2). At concentrations as low as 50 μg/ml, *E. purpurea *extract evokes a transient increase in [Ca^2+^]_i_; the response saturates at about 200 μg/ml, with an EC_50 _of 98 ± 7 μg/ml (n = 3). Transient [Ca^2+^]_i _increases were also induced with similarly prepared extracts from *E. pallida*, *E. angustifolia *and *E. tennesseensis *(data not shown).

### Evaluation of potential constituents that induce the [Ca^2+^]_i _response

**Figure 3:**
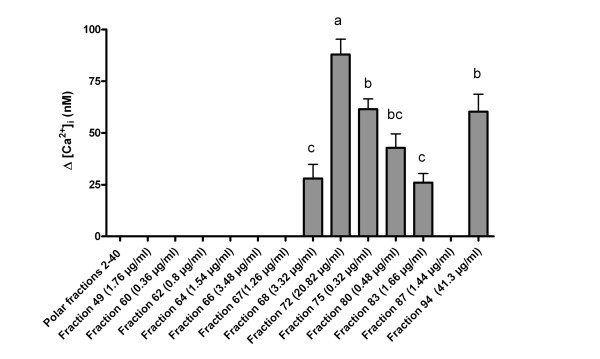
**Fractionation by preparative HPLC of the Ca^2+^-inducing activity in *E. purpurea *root ethanol extracts**. HPLC-separated fractions from 95% ethanol extract of *E. purpurea *root induce significantly different levels of Ca^2+ ^response. Fraction numbers refer to the time (in minutes) when each fraction eluted from the HPLC column. Bar graphs show the mean values of the transient increase in cytosolic Ca^2+ ^concentration evoked by different fractions (mean ± SEM, n = 3, from three independent experiments). There was no detectable response from fractions #2-67 and fraction #87. Different letters indicate statistically significant differences (p < 0.05) between the cytosolic Ca^2+ ^increase of the six active fractions by one-way analysis of variance followed by the Tukey test.

**Figure 4:**
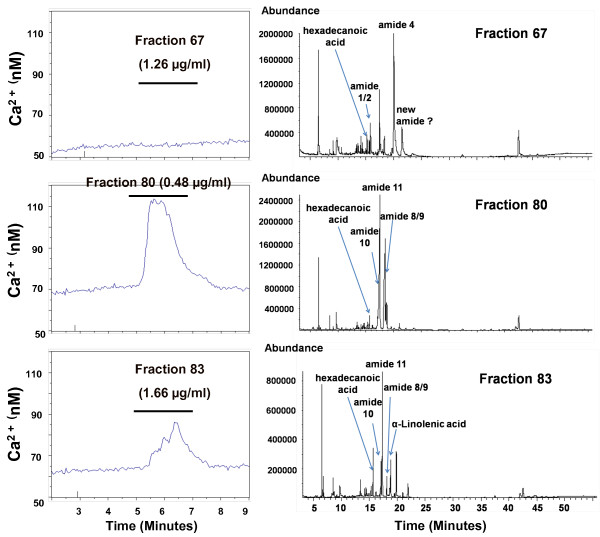
**Different HPLC-separated fractions of *E. purpurea *root ethanol extract produce different effects on transient increase in the concentration of cytosolic Ca^2+ ^in HEK293 cells**. Three example traces are shown. (Statistical analysis of data from all fractions is presented in Figure 3.) Left: Average traces from the treatment of at least 20 cells per experiment, representative of three independent experiments. Fractions were applied according to their effects in the initial trials. For each of the bioactive fractions, the increase in cytosolic Ca^2+ ^concentration was statistically significant as compared to 1% DMSO control treatment (p < 0.001). Right: GC-MS chromatograms of metabolites in these three fractions; chemically identifiable metabolites are labelled. Inactive fractions (e.g., fraction #67), were also applied after active fractions to confirm that the particular HEK293 culture used was indeed responsive to *Echinacea *extracts. The exposure time to each fraction is 2 min.

To begin identifying the constituent(s) responsible for inducing this transient increase of cytosolic [Ca^2+^]_i_, *E. purpurea *extracts were fractionated using preparative HPLC. Twenty-eight fractions were collected and tested for bioactivity. Aliquots of the HPLC fractions were applied to HEK293 cells at levels proportional to their concentrations in the initial ethanol extract. In this HPLC protocol, phenolics such as cichoric acid and cholorogenic acid elute in the more polar fractions (retention times of about 2-40 min), whereas Bauer alkamides 1, 2, 3, 4, 8, 9, 10, 11 and Chen alkamide elute in the later less polar fractions (retention times of 49-94 min) [3]. Six of the *E. purpurea *fractions (fractions #68, #72, #75, #80, #83 and #94) are active in evoking [Ca^2+^]_i _elevation in HEK293; the other 22 fractions have no detectable bioactivity (Figure 3). Both the duration and intensity of the transient [Ca^2+^]_i _increase are unique to each bioactive fraction (Figure 4). Among the six active fractions, fraction #72 has the highest activity based on the peak height of intracellular calcium concentration (Figure 3).

The constituents of each HPLC fraction were fingerprinted by GC-MS; three of these are shown in Figure 4. In addition to numerous non-alkamide constituents, fraction #68 contains Bauer alkamides 1, 2, 4, 6 and Chen alkamide; fraction #72 contains Bauer alkamides 4, 8/9, 10 and Chen alkamide; fraction #75 contains Bauer alkamides 8/9 and 10; fractions #80 and #94 contain Bauer alkamides 8/9, 10 and 11 and fraction #83 contains Bauer alkamides 8/9 and 11 (Table 1).

**Table 1:** GC-MS analysis of identified compounds in the 6 bioactive fractions of *E. purpurea *root extract ^a^.

	Fraction 68		Fraction 72		Fraction 75		Fraction 80		Fraction 83		Fraction 94	
**Compound**	**% by Dry Weight**	**μM**	**% by Dry Weight**	**μM**	**% by Dry Weight**	**μM**	**% by Dry Weight**	**μM**	**% by Dry Weight**	**μM**	**% by Dry Weight**	**μM**
**Bauer** **alkamide 1+2**	2.8	0.41	Nd	Nd	Nd	Nd	Nd	Nd	Nd	Nd	Nd	Nd
**Chen** **alkamide**	11.5	1.56	2.8	2.38	Nd	Nd	Nd	Nd	Nd	Nd	Nd	Nd
**Bauer** **alkamide 10**	Nd	Nd	1.2	1	15.1	0.19	0.4	0.01	Nd	Nd	0.8	1.33
**Bauer** **alkamide 11**	Nd	Nd	Nd	Nd	Nd	Nd	6.7	0.13	11.2	0.74	6.5	10.7
**Bauer** **alkamide 8+9**	Nd	Nd	41.9	35.32	46.9	0.61	2.5	0.05	4.6	0.31	1.8	3.01
**Bauer** **alkamide 4**	11.5	1.56	Nd	Nd	Nd	Nd	Nd	Nd	Nd	Nd	Nd	Nd
**Bauer** **alkamide 6**	5.1	0.66	Nd	Nd	Nd	Nd	Nd	Nd	Nd	Nd	Nd	Nd

^a ^The chemical identification of these alkamides in this *E. purpurea *accession, and the systematic chemical nomenclature for these alkamides have been previously described [3]. All identified constituents were confirmed via GC-MS with synthetic standards. Nd: not detectable.

Synthesized standards of Bauer alkamides 8, 10, 11, and Bauer ketone 23 were tested for bioactivity in the intracellular Ca^2+ ^assay. Bauer alkamide 11 was of particular interest because it has been reported by Raduner et al. [6] to bind to the cannabinoid receptor, CB2. None of these pure compounds display detectable bioactivity on HEK293 when applied individually, and even when applied at concentrations up to 8-fold higher than their concentrations found in the *E. purpurea *extracts (data not shown). Taken together, these results indicate that lipophilic constituents of yet unidentified structures are associated with the induction of [Ca^2+^]_i _increase in HEK293 cells by *Echinacea*. These responsible bioactive constituent(s) could be novel or alternately they might be identified in other plant species but not yet found in *E. purpurea; *for example, in *E. pallida *non-polar ketones such as pentadeca-(8 Z,13 Z)-dien-11-yn-2-one have been recently identified in *E. pallida *[14].

### *Echinacea*-induced [Ca^2+^]_i _increases in HEK293 cells appear to be associated with release of Ca^2+ ^from IP_3_-sensitive intracellular stores, and this process may involve PLC activation

**Figure 5:**
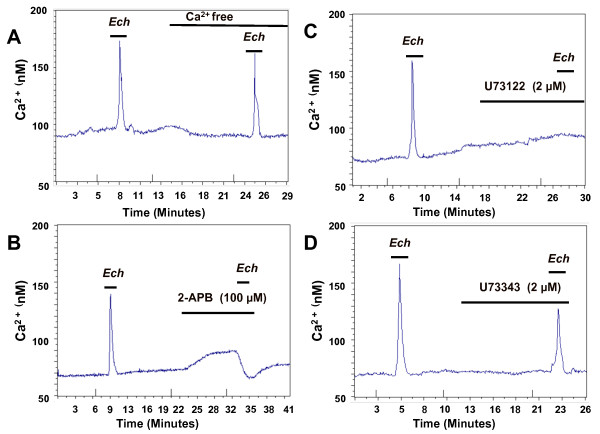
**Transient increase in cytosolic Ca^2+ ^concentration in HEK293 cells induced by *E. purpurea *root ethanol extracts is associated with Ca^2+ ^release from the IP_3_-sensitive intracellular store and the PLC pathway**. (A) Kinetic changes of [Ca^2+^]_i _in HEK cells in response to repeated applications of *Echinacea *extract (100 μg/ml) in normal (2 mM Ca^2+^) and Ca^2+^-free (EDTA-chelated) solution. Removal of Ca^2+ ^from media does not affect the *Echinacea*-evoked response when compared to the control (without Ca^2+ ^removal) (p < 0.001), indicating the response may be associated with the release of internal Ca^2+ ^stores; (B) Transient increase in cytosolic Ca^2+ ^concentration evoked by *Echinacea *can be abolished by 10 min pretreatment of cells with 100 μM of 2-APB, an IP_3 _receptor antagonist; this effect was significant (p < 0.001) as compared to the control (without pretreatment by 2-APB); (C) Transient increase in cytosolic Ca^2+ ^concentration evoked by *Echinacea *can be abolished by a 10 min pretreatment of cells with 2 μM U73122, a PLC inhibitor, indicating PLC may be required for the response; this effect is significant (p < 0.001) as compared to the control (without treatment by U73122). (D) Transient increase in cytosolic Ca^2+ ^concentration evoked by *Echinacea *cannot be abolished by a 10 min pretreatment with 2 μM U73343, an inactive analog of U73122. The effect of U73343 was not significant (p > 0.05) when compared to the control (without treatment of U73343). *Echinacea *treatment time in this set of experiments was 2 mins. Data are average traces of at least 20 cells in one experiment representative of three independent experiments.

Two principal sources of Ca^2+ ^affect the concentration of cytosolic Ca^2+^: internal Ca^2+ ^stores, primarily in the endoplasmic reticulum (ER), and extracellular Ca^2+^. To examine whether the observed *Echinacea-*induced transient [Ca^2+^]_i _increase depends on external calcium, HEK293 cells were perfused either with HEPES solution supplemented with normal concentrations of calcium (2 mM) or with EDTA-chelated calcium-free HEPES buffer for 10 min, before treatment with *E. purpurea *extracts. In both of these sets of experiments the *Echinacea-*induced transient increase in [Ca^2+^]_i _was observed (Figure 5A). Therefore, the source of the transient [Ca^2+^]_i _increase in the *Echinacea*-treated HEK293 cells appears to be from intracellular stores, as indicated by the stimulatory effect that occurs despite the cells being in calcium-free media.

Release of Ca^2+ ^from internal ER-stores typically occurs via an inositol-1,4,5-trisphosphate (IP_3_) receptor, however, other mechanisms exist as well [15]. We tested for the possible involvement of the IP_3 _receptor in the *Echinacea-*induction of intracellular Ca^2+ ^release by evaluating the effect of 2-aminoethoxydiphenyl borate (2-APB), an IP_3 _receptor antagonist, on *Echinacea-*induced cytosolic Ca^2+ ^increase. If *Echinacea*-extracts induce intracellular Ca^2+ ^via an IP_3 _receptor, blocking this receptor should eliminate the *Echinacea-*induced increase in [Ca^2+^]_i_. 2-APB (100 μM) completely abolishes the transient increase in [Ca^2+^]_i _evoked by the *Echinacea*-extract (Figure 5B), consistent with the role of the IP_3 _receptor in mediating bioactivity.

To test whether phospholipase C (PLC) activation is required for the *Echinacea-*induced increase in [Ca^2+^]_i_, the effects of U-73122, a specific PLC inhibitor, and its inactive analog U-73343 were evaluated. In these studies, the HEK293 cell cultures were treated with *Echinacea *extracts first, to show that the cultures were indeed able to respond to *Echinacea*. The PLC antagonist, or its inactive analog, was then applied for 10 min and the *Echinacea *extract was reapplied to test whether there are any blocking effects of the antagonist. Application of U-73122 completely abolishes the increase in [Ca^2+^]_i _that is evoked by the *Echinacea*-extract (Figure 5C), whereas U-73343 has no such effect (Figure 5D), consistent with an involvement of the PLC pathway.

### Thapsigargin and 2-ABP inhibition experiments indicate that *Echinacea *extract may induce Ca^2+ ^influx by activating SOCE following depletion of Ca^2+ ^from intracellular stores

**Figure 6:**
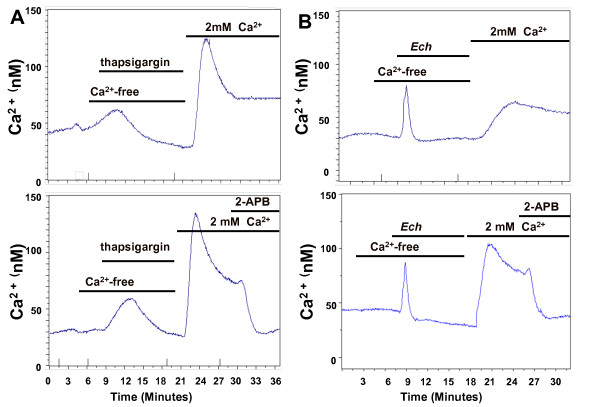
***Echinacea *-evoked depletion of Ca^2+ ^from intracellular stores induced Ca^2+ ^influx via SOCE in HEK293 cells**. This response is pharmacologically similar to the Ca^2+ ^influx evoked by thapsigargin. (A) Top: After depletion of Ca^2+ ^stores by treating cells with 2 μM thapsigargin in Ca^2+^-free conditions for 10 min, 2 mM Ca^2+ ^was added to assess SOCE; Bottom: Ten min after restoration of external Ca^2+^, 100 μM 2-APB was applied for 10 mins; (B) Top: After depletion of Ca^2+ ^stores by treating cells with *Echinacea *extract (100 μg/ml) in Ca^2+^-free conditions for 10 min, 2 mM Ca^2+ ^was added to assess SOCE; Bottom: Ten min after restoration of external Ca^2+^, 100 μM 2-APB was applied for 10 mins. Data shown are average traces of at least 20 cells in one experiment representative of three independent experiments.

To experimentally evaluate whether store operated calcium entry (SOCE) plays a role in calcium homeostasis in the HEK293 cell response to *Echinacea*-extracts, external Ca^2+ ^was removed and internal Ca^2+ ^stores were depleted using the sarcoplasmic/ER Ca^2+ ^-ATPase (SERCA) pump inhibitor, thapsigargin. Restoration of external Ca^2+ ^following thapsigargin-treatment indicates the function of SOCE, which can be observed as an increase in cytosolic Ca^2+ ^[16]. Here, the effect of thapsigargin is compared to the effect of *Echinacea *extract. The Ca^2+ ^responses evoked by *Echinacea *and thapsigargin are similar (Figure 6). In the absence of extracellular Ca^2+^, both agents evoke a transient intracellular Ca^2+ ^elevation, interpreted as being due to Ca^2+ ^release from internal stores. After Ca^2+^-containing medium is introduced, a sustained increase in cytosolic Ca^2+ ^is observed for both treatments (Figure 6A, 6B). This pattern is consistent with SOCE-mediated changes in the concentration of cytosolic Ca^2+ ^[16].

To examine whether thapsigargin- and *Echinacea*-induced Ca^2+ ^entry pathways are pharmacologically similar in the HEK model, 2-APB was applied in the continued presence of external Ca^2+^. 2-APB is considered the most potent and consistent compound in blocking SOCE, acting as a potent SOCE inhibitor independent of, and in addition to, its ability to inhibit the IP_3 _receptor [17]. 2-APB has no effect on voltage-operated Ca^2+ ^channels [18] or on non-voltage-activated Ca^2+ ^entry pathways [19]. Therefore, despite its dual effects, 2-APB provides a critical reagent for investigating SOCE activation and for discriminating between different forms of Ca^2+ ^entry [17]. 100 μM 2-APB completely blocks SOCE induced by both thapsigargin and *Echinacea *extracts (Figure 6C, 6D). This result is consistent with the involvement of an SOCE pathway in the Ca^2+ ^elevation associated with *Echinacea*-induced depletion of Ca^2+ ^from intracellular stores.

### CB2 is not involved in *Echinacea*-induced [Ca^2+^]_i _increase in HEK293 cells

**Figure 7:**
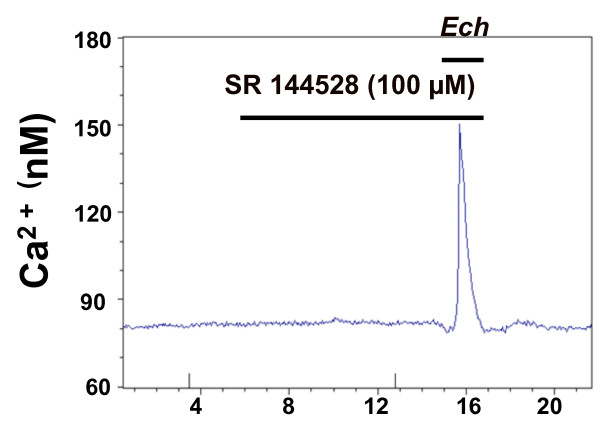
**Transient increase in cytosolic Ca^2+ ^concentration evoked by *Echinacea *extracts is not mediated by CB2 receptor activation**. *Echinacea *extract (100 μg/ml)-evoked cytosolic Ca^2+ ^transients in HEK293 cells (exposure time was 2 mins) cannot be abolished by a 10 min pretreatment with the specific CB2 receptor antagonist SR 144528 (100 μM). The effect of SR 144528 was not significant (p > 0.05) when compared to the control (without treatment of SR 144528). Data are average traces of at least 20 cells in one experiment representative of three independent experiments.

CB2 was not detected in HEK293 cells in multiple microarray studies of HEK293 [10]; over 200 public microarray data in ArrayExpress at http://www.ebi.ac.uk/microarray-as/ae/]. Due to a mounting interest in CB as a potential target for *Echinacea *action [6-8], we also tested the ability of SR 144528, a specific CB2 receptor antagonist, to effect the *Echinacea*-induced [Ca^2+^]_I _increase. In these studies, SR 144528 (100 μM) was applied for 10 min and then the *Echinacea *extract (100 μg/ml) was applied to test whether there are any blocking effects of the antagonist. Our results showed that SR 144528 was not able to abolish the *Echinacea *response (Figure 7). This confirmed that CB2 was indeed not responsible for the *Echinacea*-induced [Ca^2+^]_i _elevation observed in HEK293 cells.

## Discussion

Despite numerous reports on physiological and cellular consequences of treatments with *Echinacea *[4,11,19], little is known about the early molecular mechanisms that might mediate these events. One possibility is that *Echinacea *acts in part via Ca^2+^, a central intracellular messenger that participates in the regulation and co-regulation of inflammation [20] and pain [21]. A recent report describes an increase in Ca^2+ ^in HL60 cells that is induced by alkamides of *Echinacea*, and mediated via the CB2 receptor [8]. Here, we describe an effect of *Echinacea *components in a non-immune-related cell type that lacks CB2 receptors. Based on these data, we reveal an *Echinacea*-induced stimulation of an increase in cytosolic Ca^2+ ^that is not-CB2-dependent, and is not associated with the major alkamides of *E. purpurea*.

**Figure 8:**
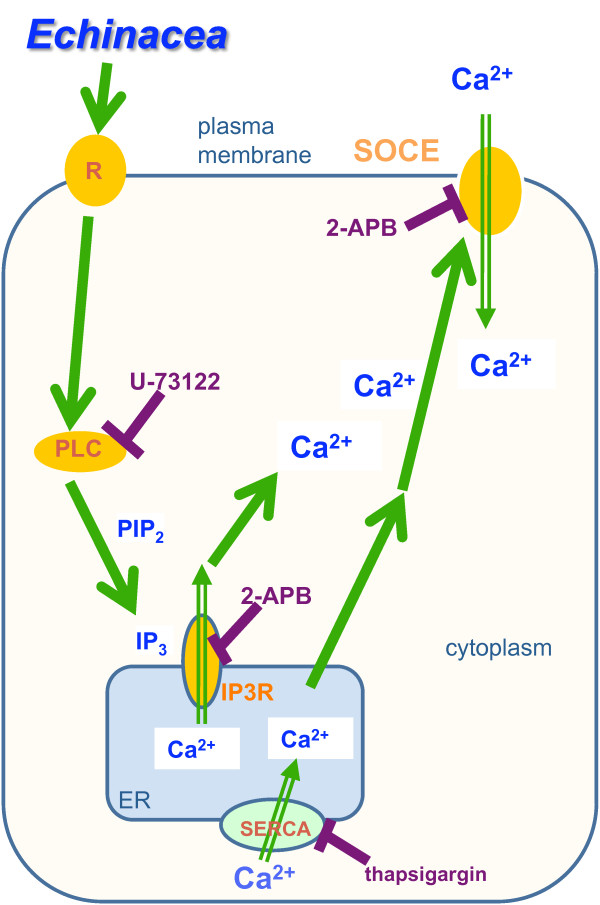
**Proposed metabotropic mechanism for Ca^2+ ^signaling in HEK293 cells underlying the modulation of some of the cellular responses induced by *Echinacea *extracts**. Green arrows indicate steps proposed from data in this paper. Purple lines indicate inhibitors used in this study. In this model, lipophilic constituents in *Echinacea *extracts bind to unidentified surface membrane receptor(s) (R), resulting in the activation of PLC. PLC-activation catalyzes the production of IP_3 _from PIP_2_. IP_3 _binds to and opens IP_3_R in the membrane of the ER, resulting in the release of Ca^2+ ^from ER Ca^2+ ^store. The decrease in the Ca^2+ ^content of the ER is sensed by STIM1, which in turn activates SOCE in the plasma membrane. Thus, the level of cytosolic Ca^2+ ^is increased through release of Ca^2+ ^from the ER Ca^2+ ^store as well as Ca^2+ ^influx via SOCE. Receptors endogenous to HEK293 cells include: chemokine receptor; muscarinic acetylcholine receptor; glutamate receptor; B2-bradykinin receptor; and P2X, P2Y purinergic receptors [10]; these represent candidates for interaction with *Echinacea *constituents. PLC: phospholipase C; IP_3_, inositol-1,4,5-trisphosphate; PIP_2_: phosphatidylinositol-4,5-bisphosphate; IP_3_R: IP_3 _receptor; ER: endoplasmic reticulum; STIM1: stromal interaction molecule 1; SOCE: store-operated calcium entry.

This *Echinacea-*induced Ca^2+ ^influx of HEK293 cells is likely associated with an IP_3 _mediated signaling pathway (Figure 8). Ca^2+ ^influx is rapid, does not require external Ca^2+ ^and is eliminated by the IP_3 _receptor antagonist, 2-APB. Furthermore, the PLC pathway may play a role in this release, as suggested by the observation that the *Echinacea*-evoked increase in cytosolic Ca^2+ ^is blocked by the PLC antagonist U-73122, but not by its inactive analog (U-73343).

A PLC-dependent signal might be mediated in several ways. For example, constituents of *Echinacea *might bind to a surface membrane receptor that is coupled to PLC, such as chemokine [22], glutamate [23], or purinergic receptors [24]. Microarray data indicate these receptors are present in HEK293 cells [10]. The observation that active fractions of the *E. purpurea *extract have differential kinetic properties and potency on HEK293 cells (e.g., bioactive HPLC-fractions #80 and #83) is consistent with more than one receptor being stimulated by *Echinacea *extracts. A second possibility is that bioactive constituent(s) enter the cell and act directly or via an intracellular moiety on PLC. These possibilities are not mutually exclusive. Because a portion of the Ca^2+ ^response that is induced by *Echinacea *extracts is associated with lipophilic constituent(s), logical candidates for being permeable across cell membranes [25], we can not exclude this possibility.

Calcium stored in the ER is released through Ca^2+ ^channels in the ER membrane, usually via the IP_3 _receptor or ryanodine receptor families [15]. Ca^2+ ^pumps located in the ER membranes then return cytosolic Ca^2+ ^into the lumen, thus contributing to Ca^2+ ^homeostasis between the cytoplasm and ER. The intracellular Ca^2+ ^stores are refilled from the extracellular reservoir, mostly through SOCE [16]. SOCE has been reported in multiple cell types, e.g., smooth muscle cells, epithelial cells, hippocampal cells, and regulates physiological processes such as inflammation, cardiac contraction, and neurotransmission [26].

Our data indicate that the source of the initial transient increase in cytosolic calcium levels that are induced by *Echinacea*-extracts is from internal stores, indicative of a metabotropic response. In this metabotropic model, PLC activation would lead to the production of IP_3_, which in turn would activate the IP_3 _receptor causing release of Ca^2+ ^from the ER [15]. The participation of the IP_3 _receptor in the *Echinacea*-induced calcium release is suggested by treatment of HEK293 cells with the specific membrane-permeable IP_3 _receptor antagonist, 2-APB [17]. 2-APB is able to completely abolish the *Echinacea*-induced calcium release from internal stores. 2-APB has been reported to enhance leakage of Ca^2+ ^from the ER and inhibit SERCA activity, resulting in enhancement of Ca^2+ ^signaling [27]. This complex action of 2-APB is consistent with the small initial intracellular calcium increase we observed in HEK293 cells after 2-APB application.

This model predicts that the *Echinacea*-induced release of Ca^2 ^from internal stores may be coupled to a subsequent activation of the SOCE process. In many cell types, depletion of intracellular Ca^2+ ^stores results in the opening of SOCE in the plasma membrane [16]. SOCE, thought to mediate aspects of cell secretion and motility, cell proliferation and gene expression by altering cellular Ca^2+ ^[16], is considered a promising target for therapeutic treatment in inflammatory diseases [28]. The nature of SOCE, and the mechanism linking Ca^2+^-store content to the opening of this Ca^2+ ^channel, remains unclear. Two proteins have been implicated in SOCE function: Orai1, a pore-forming subunit of the SOCE, and stromal interaction molecule 1 (STIM1), thought to be an ER-based Ca^2+ ^sensor that activates SOCE by an as yet undefined mechanism [16]. Therefore we propose that the resultant decrease of Ca^2+ ^in ER after *Echinacea *treatment would in turn activate the plasma membrane SOCE through a mechanism that involves STIM1. Taken together, this model predicts that in HEK293 cells, the level of cytosolic Ca^2+ ^associated with *Echinacea *treatment increases through two mechanisms: initially the release of Ca^2+ ^from ER Ca^2+ ^stores, and subsequently Ca^2+ ^influx via SOCE.

The physiological events downstream of a cytosolic Ca^2+ ^increase, whether *Echinacea*-induced or otherwise, are complex and highly dependent on the cell type and context in which they occur. Longer range effects of changes in cytosolic levels of Ca^2+ ^regulate a wide variety of cellular processes [20]. In T-cells, for example, elevated intracellular Ca^2+ ^activate Ca^2+^-dependent enzymes, such as calcineurin, and thereby transcription factors, such as nuclear factor of activated T cells (NFAT) and nuclear factor-κB (NF-κB). These transcription factors modulate the activation of T-cells and generation of cytokines, which in turn regulate the expression of many target genes in inflammation and pain transmission [20].

Studies using animal and human models indicate that *Echinacea *extracts enhance the cyclooxygenase 2 and cytokine signaling activities of various immune cells, both of which are involved in many steps of immunomodulatory responses and mediation of pain transmission [4,11,19]. Consistent with this concept, microarray analyses indicate that *Echinacea *preparations modulate the levels of varied cytokine transcripts in human acute monocytic leukemia cells, bronchial epithelial cells and dendritic cells [29-31]. The overall transcript profiles in these microarray experiments are diverse, although it is not clear as to whether this variation is associated with the use of different cell models or different *Echinacea *preparations. Taken together, these studies and our own convey the important message that *Echinacea *may induce many responses in various cell types involving more than one signaling pathway, and that it is a combination of these responses that likely lead to the overall physiological effect on the organism.

This report highlights the effect of lipophilic, non-alkamide *Echinacea *components in a non-immune-related cell type that lacks CB2 receptors. Our use of a human non-immune cell line as an experimental system to evaluate *Echinacea*-induced response emphasizes the complex effects of herbal medicines, and sheds more light on potential molecular early signaling mechanisms for this important medicinal plant. Thus the non-CB-related intracellular calcium signaling induced by non-alkamide components of *Echinacea *extracts revealed in this study, in conjunction with the activation of CB-mediated signaling by *Echinacea *extracts by specific alkamides [6-8], provides an intriguing example of how the chemical complexity of a single medicinal species can affect diverse signaling receptors and pathways in a cell-type dependent manner.

## Conclusions

In conclusion, we show that extracts from *Echinacea *induce transient increases of cytosolic Ca^2+ ^levels when applied to HEK293 cells, and that this increase can be attributed to a subset of lipohilic *Echinacea *constituents. HPLC fractionation reveals six distinct lipophilic fractions that induce this transient increase in cytosolic Ca^2+^. This bioactivity does not appear to be attributable to any known bioactive components of *Echinacea*, including the alkamides. Furthermore, it is not associated with the CB receptors. Based on studies with a range of inhibitors, activators and experimental conditions, we propose that *Echinacea *extract contains compounds other than its known alkamides that induce cytosolic Ca^2+ ^release, in combination with an ER-depletion-associated activation of the SOCE pathway in HEK293 cells.

## Competing interests

The authors declare that they have no competing interests.

## Authors' contributions

LW carried out the study design, experimental work on cell culture and calcium imaging data and conducted statistical analyses, literature search and drafted the manuscript. EWR participated in design and coordination of the study and provided assistance in data interpretation. KJ helped with the calcium imaging work. SJ provided assistance in data interpretation. LR did the GC-MS analysis of the plant extract and fractions. BJN evaluated the data and corrected the manuscript for publication. JM helped with the cell culture. MK provided assistance in data interpretation and edited the manuscript. ESW conceived the study, supervised the experimental work and co-wrote the manuscript. All authors read and approved the final manuscript.

## Pre-publication history

The pre-publication history for this paper can be accessed here:

http://www.biomedcentral.com/1472-6882/10/72/prepub
